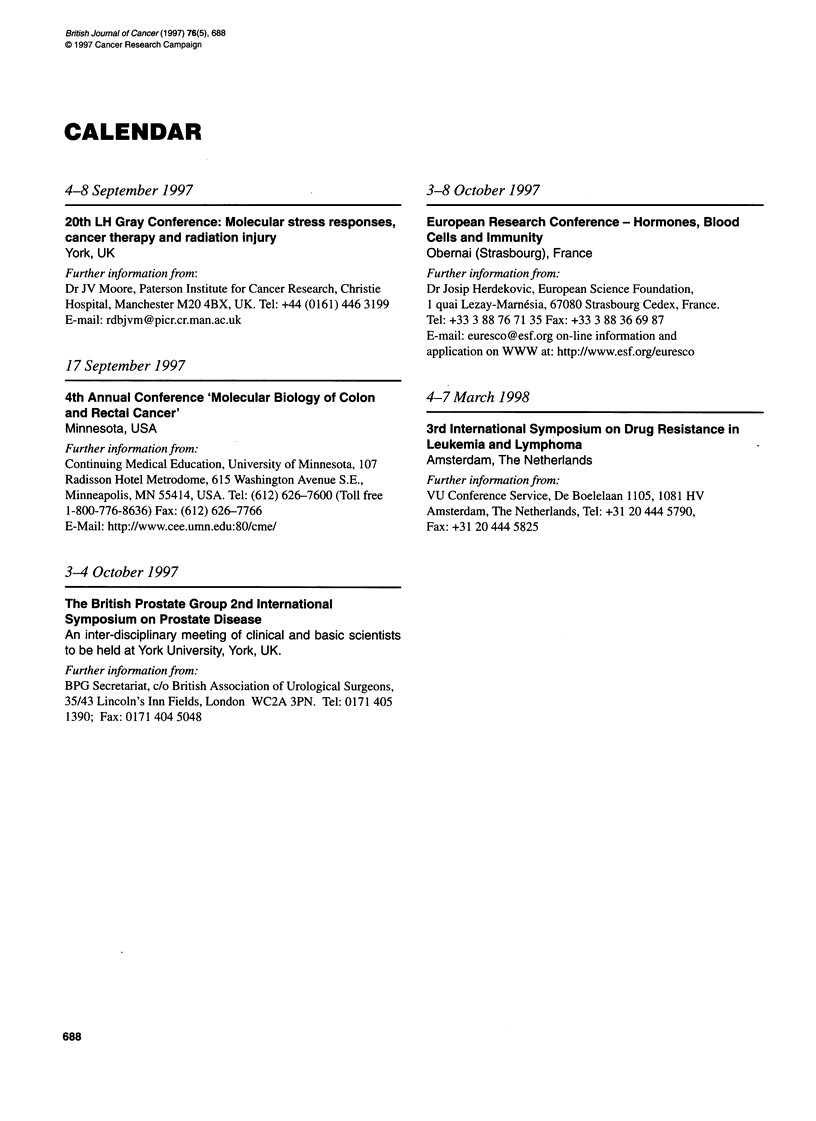# Calendar

**Published:** 1997

**Authors:** 


British Journal of Cancer (1997) 76(5), 688
© 1997 Cancer Research Campaign

### CALENDAR

*4–8 September 1997*

**20th LH Gray Conference: Molecular stress responses, cancer therapy and radiation injury**
York, UK

*Further information from:*
Dr JV Moore, Paterson Institute for Cancer Research, Christie Hospital, Manchester M20 4BX, UK. Tel: +44 (0161) 446 3199
E-mail: rdbjvm@picr.cr.man.ac.uk

*17 September 1997*

**4th Annual Conference 'Molecular Biology of Colon and Rectal Cancer'**
Minnesota, USA

*Further information from:*
Continuing Medical Education, University of Minnesota, 107 Radisson Hotel Metrodome, 615 Washington Avenue S.E., Minneapolis, MN 55414, USA. Tel: (612) 626–7600 (Toll free 1-800-776-8636) Fax: (612) 626–7766
E-Mail: http://www.cee.umn.edu:80/cme/

*3–4 October 1997*

**The British Prostate Group 2nd International Symposium on Prostate Disease**
An inter-disciplinary meeting of clinical and basic scientists to be held at York University, York, UK.

*Further information from:*
BPG Secretariat, c/o British Association of Urological Surgeons, 35/43 Lincoln's Inn Fields, London WC2A 3PN. Tel: 0171 405 1390; Fax: 0171 404 5048

*3–8 October 1997*

**European Research Conference – Hormones, Blood Cells and Immunity**
Obernai (Strasbourg), France

*Further information from:*
Dr Josip Herdekovic, European Science Foundation, 1 quai Lezay-Marnésia, 67080 Strasbourg Cedex, France.
Tel: +33 3 88 76 71 35 Fax: +33 3 88 36 69 87
E-mail: euresco@esf.org on-line information and application on WWW at: http://www.esf.org/euresco

*4–7 March 1998*

**3rd International Symposium on Drug Resistance in Leukemia and Lymphoma**
Amsterdam, The Netherlands

*Further information from:*
VU Conference Service, De Boelelaan 1105, 1081 HV Amsterdam, The Netherlands, Tel: +31 20 444 5790,
Fax: +31 20 444 5825